# Breath of Change: Evaluating Asthma Information on TikTok and Introducing the Video Health Information Credibility Score

**DOI:** 10.7759/cureus.54247

**Published:** 2024-02-15

**Authors:** Bilal Irfan, Ihsaan Yasin, Aneela Yaqoob

**Affiliations:** 1 Microbiology and Immunology, University of Michigan, Ann Arbor, USA; 2 Electrical Engineering and Computer Science, University of Michigan, Ann Arbor, USA; 3 Infectious Diseases, Beaumont Hospital, Taylor, USA

**Keywords:** social media analytics, digital health education, tiktok, asthma, online patient education, vhics

## Abstract

Introduction

Asthma’s global prevalence underscores the need for accessible health information dissemination, especially in the digital age. TikTok, known for its wide reach and diverse content, presents both opportunities and challenges in health information dissemination. This study aims to characterize the quality and reach of asthma-related content on TikTok and introduces the Video Health Information Credibility Score (VHICS) as a novel tool for quality assessment.

Materials and methods

We used a systematic methodology to analyze the top 100 TikTok videos by the number of likes tagged with #asthma. Data were collected in June 2023 and January 2024 to allow for temporal trend analysis. Videos were evaluated based on engagement metrics (views, likes, comments, shares, and favorites) and quality using the DISCERN instrument.

Results

Our analysis showed that physician-generated content accounted for a significant proportion of asthma-related videos, with varying levels of engagement. The DISCERN scores, with a range of 1 (lowest) to 5 highest), provided insights into content quality, revealing trends in user engagement and information reliability over time. Temporal analysis indicated changes in content creation and audience interaction.

Discussion

The study highlights the evolving landscape of digital health communication on TikTok. The introduction of VHICS added depth to the quality assessment of future directions, indicating the necessity for accurate and reliable health information on social media. The findings suggest an imperative for healthcare professionals to address misinformation and leverage digital platforms for patient education effectively.

Conclusions

TikTok is a significant medium for health information dissemination, with substantial potential for impact in patient education. The introduction of VHICS can enrich the analysis of video content, offering a robust tool for assessing the quality of health information on social media. This study underscores the importance of credible, clear, and audience-relevant health communication in the digital era.

## Introduction

Asthma, which is often characterized as a chronic inflammatory disorder of the bronchial mucosa, can lead to repeated episodes of symptomatic display, including feelings of breathlessness, tightness in the chest, coughing, or wheezing [1]. Its occurrence during the early morning hours and night times can be attributed to a range of physiological patterns, including low plasma cortisol levels as part and parcel of the normal circadian variation [2,3]. The condition that is polygenic in nature is stated to arise from the interaction between various genetic and environmental factors, which can lead to airway hyperresponsiveness and airflow obstruction [4]. A 2019 study points to around 262 million cases of asthma arising, a notable decrease from four years prior, when there were 358 million reported cases [5]. 

The pathophysiology of asthma involves a multitude of factors in regard to the synthesis of environmental stimuli and early childhood infections with underlying genetic impulses. The chronic inflammation responsible for the disorder is caused by the activation of eosinophils, T lymphocytes, mast cells, neutrophils, and dendritic cells [6]. These cells release a range of mediators, such as histamines, leukotrienes, and cytokines, which perpetuate the inflammatory response [7]. Th-2 lymphocyte-predominant immune responses are developed, and thereafter, chronic airway changes occur, which may contribute to gradual airflow obstruction [8]. These are important considerations for clinicians and policy makers to have as the global prevalence of asthma underscores the urgent need for accessible, reliable, and relevant health information dissemination. Historically, health awareness campaigns, brochures, and physician-patient interactions were primary sources of such information [9]. Nevertheless, the advent of the digital era has metamorphosed the landscape of health information dissemination, particularly in the realm of social media platforms that remain unparalleled in reach and immediacy, thus establishing themselves as powerhouses in this transformation [10]. 

TikTok is one such platform characterized by the spread of short videos by content creators from across different backgrounds, and it has witnessed exponential growth since its inception [11]. Its algorithmically driven content presentation, coupled with the platform’s global reach, positions it uniquely in the realm of health information dissemination [12]. Given this backdrop, it is imperative to characterize the quality and reach of asthma-related content on this platform. The dynamism of TikTok not only offers opportunities for health professionals to engage with diverse populations but also presents challenges concerning the accuracy and reliability of disseminated content. In response to these challenges, this study introduces the “Video Health Information Credibility Score” (VHICS), a novel tool designed to quantitatively assess the credibility, clarity, and relevance of health information presented in TikTok videos. The VHICS tool, akin to the DISCERN scoring system but specifically tailored for video content, will be utilized to evaluate asthma-related videos on TikTok, providing a structured method to gauge their informational quality.

In this study, our overarching objective is to elucidate the landscape of asthma-related content on TikTok. By dissecting trends, gauging information quality, and understanding audience engagement metrics, we aim to provide insights that could pave the way for more effective, accurate, and impactful health communication strategies on digital platforms in the future.

## Materials and methods

In the course of this study, we meticulously crafted a systematic methodology to identify and scrutinize TikTok videos that propagated asthma-related content. The overarching goal was to achieve a profound understanding of the nature, origin, and public interaction with this content, upholding the study’s scientific precision and relevance. To categorize and assess this vast content, we initiated our approach by targeting the hashtag #asthma on TikTok. Leveraging hashtags is a well-acknowledged tactic in social media research, given their role as categorical markers of content themes [13,14]. To circumvent temporal inconsistencies and to provide a more static analysis, our data were extracted on June 5, 2023, providing a snapshot of the prevailing asthma-related content. In addition to this initial data collection, a subsequent extraction was conducted on January 5, 2024. This additional data capture allowed us to perform a temporal trend analysis, comparing the engagement and quality metrics over an eight-month period to identify any significant changes or trends in the asthma-related content on TikTok.

In a bid to set clear boundaries for our analysis, we established criteria for video selection. Videos needed to be among the top 100 ranked by “likes,” a criterion that ensured we honed in on the most engaging content. We steered clear of videos not in English, duplicates, and those that strayed from pertinent asthma-centric discourse. To ensure a comprehensive understanding of the evolving trends, the same selection criteria were applied during the second extraction phase in January 2024. This approach allowed for a consistent and comparable analysis of the content over time, with 100 videos being analyzed in June 2023 and another 100 in January 2024, for a total of 200.

Further granularity was achieved by organizing the data on account of a variety of demographic factors. Content creator profiles were looked at to divulge information on professional backgrounds, ranging from physicians, nonphysicians, private companies, or other health professionals. Further stratification was carried out for videos generated by physicians, distinguishing between pulmonologists and other specialties. Additionally, a thematic analysis enabled us to categorize content by the nature of the message conveyed, be it advertising, home remedies, educational content, or personal experience. This detailed classification of content was pivotal in assessing the diversity and range of asthma-related information available on the platform.

We logged metrics indicative of user engagement to gauge the actual impact and resonance of these videos. These encompassed parameters like views, likes, comments, shares, and favorites, which collectively provide a composite view of a video’s reach and popularity. Central to our analysis was the quality of the content, which we evaluated using the renowned DISCERN instrument. Typically reserved for written health material, this instrument was adapted to suit video content for our study [15]. A dual-reviewer system ensured that each video underwent rigorous scrutiny. Any discrepancies between the reviewers were addressed in consensus meetings, and averages of the two scores were reported for a given video, harmonizing the assessment process. Furthermore, to quantify and analyze the temporal shifts in content quality and audience engagement, statistical tests such as regression analysis and t-tests were employed. These tests provided a more nuanced understanding of the relationship between the DISCERN scores and engagement metrics over the two time points, thereby enhancing the robustness of our findings. Ethical considerations were not overlooked; although specific ethical sanctions were not requisitioned due to the public nature of TikTok content, we remained vigilant, anonymizing data and honoring the rights of content creators and platform-specific guidelines.

## Results

Upon rigorous evaluation of the curated dataset, salient patterns and trends in asthma-related content on TikTok emerged. Across the analyzed videos, physicians were the principal content creators for 35% of the videos. These physician-led videos garnered an average viewership of 84,286, with the DISCERN instrument reflecting a mean score of 1.201. In contrast, non-physician creators, accounting for 31% of the content, exhibited a substantial viewership spike with an average of 128,338 views. Intriguingly, despite the higher viewership, the mean DISCERN score for non-physicians stood at 1.234, only marginally higher than their physician counterparts (Table 1). Regression analysis evaluates the association between engagement metrics and the DISCERN score.

**Table 1:** Overview of asthma content on TikTok in June 2023

	Percentage of videos	Average views	Average likes	Average comments	Average shares	Average favorites	DISCERN
Profession							
Physician	35	84,286	4189	179	1078	1375	1.201
Non-physician	31	128,338	17,328	213	913	1403	1.234
Private company	19	355,369	25,786	994	1814	3402	1.194
Other health Profession	15	179,487	6253	167	1225	1956	1.191
Sex							
Male	65	395,573	56,642	783	5432	10753	1.203
Female	32	197,544	21,713	396	2287	6067	1.241
Other designation	3	154,388	23,053	994	1814	5022	1.194
Physician specialty							
Pulmonologists	24	185,323	5967	252	1563	1991	1.186
Non-pulmonologists	11	150,423	311	22	23	30	1.236
Experience							
Advertising	29	189,634	19,035	348	1134	2404	1.197
Home Remedies	16	119,820	3935	88	531	687	1.225
Educational content	35	198,183	10,930	597	1334	1351	1.211
Personal experience	20	181,594	13,500	94	1543	2881	1.209

Private companies, comprising 19% of the content, presented an even more pronounced viewership, averaging a staggering 355,369 views. However, the quality assessment via the DISCERN instrument yielded a score of 1.194, subtly hinting at a potential compromise in content quality. Other health professionals contributed to 15% of the content with an average view count of 179,487 and a DISCERN mean score closely aligned with private entities at 1.191.

**Figure 1:**
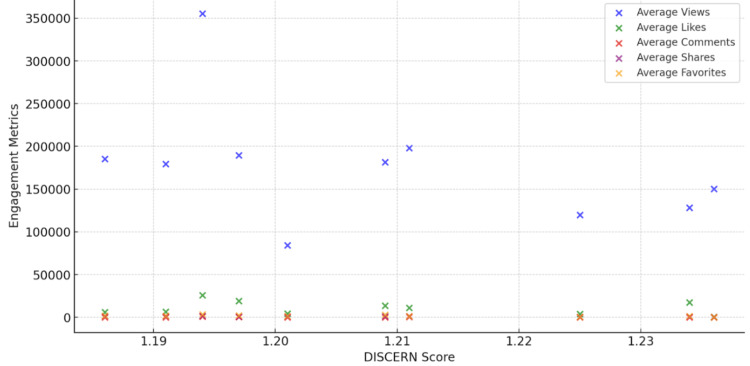
Correlation between DISCERN score and engagement metrics

Pulmonologists who are recognized experts in the realm of asthma generated nearly a quarter of the overall tabulated content, securing an average viewership of 185,323 and a DISCERN mean score of 1.186 [16]. In comparison, non-pulmonologist physicians, accounting for 11% of the videos, obtained an average view count of 150,423 and a DISCERN score of 1.236. Thematic content analysis showcased that educational content (35%) and personal experiences (20%) dominated the platform. These categories, while different in nature, both demonstrated high engagement metrics, underscoring the TikTok community’s penchant for informative and relatable content (Figure 1).

**Figure 2:**
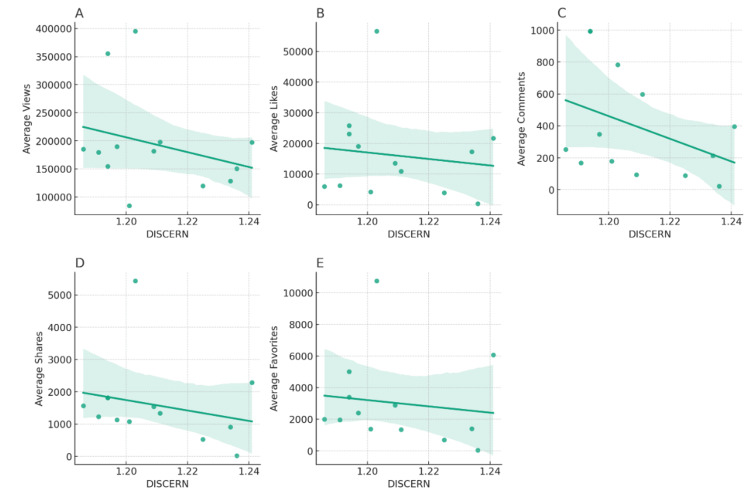
Regression analysis between engagement metrics and DISCERN score (A) Regression analysis for DISCERN score and average views. (B) Regression analysis for DISCERN score and average likes. (C) Regression analysis for DISCERN score and average comments. (D) Regression analysis for DISCERN score and average shares. (E) Regression analysis for DISCERN score and average favorites.

A regression analysis examining the relationship between the DISCERN score and various engagement metrics reveals some interesting trends (Figure 2).

The regression equations describe the relationship, while the R^2^ values indicate the strength of this relationship, with a higher R^2^ value signifying a stronger relationship (Table 2). 

**Table 2:** Regression equations for respective engagement metrics

Engagement metric	Regression equation	R^2^ value	Interpretation
Average views	y = -1770057.59x + 2316183.29	0.1912	Moderate negative relationship with DISCERN score
Average likes	y = -106255.90x + 139123.03	0.0557	Weaker relationship with DISCERN score
Average comments	y = -6569.05x + 8233.44	0.1605	Moderate negative relationship with DISCERN score
Average shares	y = -22833.93x + 28708.32	0.6020	Strong negative relationship with DISCERN score
Average favorites	y = -38603.78x + 48396.81	0.4772	Relatively strong negative relationship with DISCERN score

**Figure 3:**
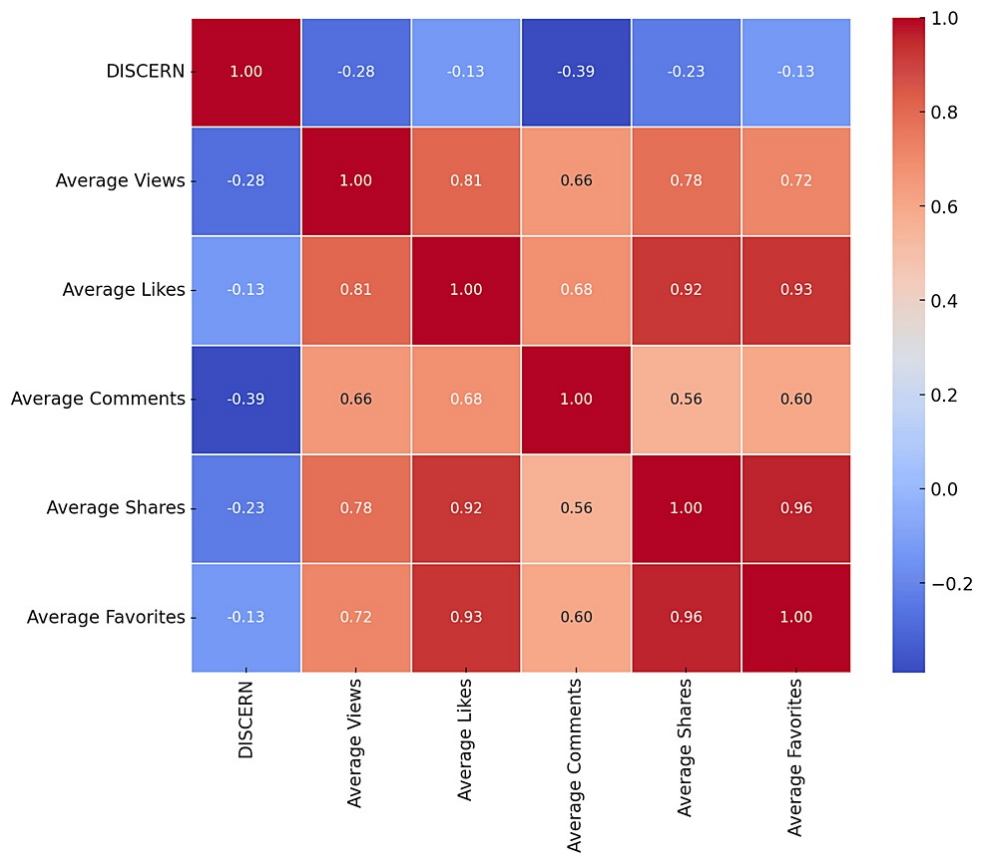
Correlation heatmap of DISCERN scores and engagement metrics

A heatmap can aid in easily identifying which engagement metrics are more closely related to the DISCERN score and each other. For example, strong colors (red or blue) indicate stronger correlations, while colors closer to white indicate weaker correlations. This can provide valuable insights into how different aspects of the content and its reception are interrelated (Figure 3).

A new search conducted in January 2024 with the #asthma revealed some changes in regard to the content metrics (Table 3).

**Table 3:** Brief overview of asthma content on TikTok in January 2024

Category	Percentage of videos	Average views	Average likes	Average comments	Average shares	Average favorites	DISCERN
Physician	38.69%	255,907.83	18,357.75	404.83	1693.26	1437.33	1.13
Non-physician	33.66%	164,248.57	18,153.17	260.82	1856.31	2611.71	1.20
Private company	20.66%	83,226.64	21,374.49	499.46	1880.91	2648.81	1.16
Other health profession	24.31%	212,812.49	9,587.09	632.26	1546.78	2088.07	1.17

The results of the t-tests conducted to compare the changes in various metrics on TikTok asthma-related content between June 2023 and January 2024 revealed no significant differences (Table 4).

**Table 4:** Statistical comparison of engagement and quality metrics for asthma-related content on TikTok (June 2023 vs. January 2024)

Metric	T-statistic	p-value	Significant difference (p < 0.05)
Views	0.11	0.91	No
Likes	-0.62	0.56	No
Comments	-0.28	0.79	No
Shares	-2.31	0.06	No
Favorites	-0.29	0.78	No
DISCERN	2.29	0.06	No

A new tool was created to aid in quantitatively analyzing TikTok videos, especially in the context of health-related content, the VHICS. This tool serves to specifically assess the credibility, clarity, and relevance of health information presented in video format. VHICS would be particularly useful for platforms like TikTok, where content is dynamic, visual, and often brief (Table 5). 

**Table 5:** VHICS calculations VHICS, Video Health Information Credibility Score

Criteria	Description	Score (1-5)
Accuracy	Accuracy of health information based on current scientific evidence	1-5
Source credibility	Credibility of the content creator (qualifications, affiliations, etc.)	1-5
Production quality	Quality of video production (clarity of visuals and audio, editing, etc.)	1-5
Information clarity	Clarity and understandability of the information presented	1-5
Balance and bias	Objectivity of content and absence of commercial or ideological bias	1-5
Relevance to audience	Relevance of content to the target audience (considering cultural sensitivity)	1-5
Engagement and interaction	Level of viewer engagement and interactive elements (e.g., Q&A and polls)	1-5
Referencing and supporting data	Presence of references to scientific data, studies, or expert opinions	1-5

The VHICS is calculated by summing the scores for each criterion and dividing by the number of criteria. A higher VHICS would indicate a video with high credibility and quality of health information.

## Discussion

The exploration of asthma-related content on TikTok has unveiled profound insights, necessitating a deeper contemplation on the interplay of healthcare information, audience preferences, and the digital revolution. At the very outset, the divergence in viewership between physician and non-physician content creators strikes a chord. Despite physicians, with their deep-rooted medical expertise, contributing to 35% of the content, non-physicians, armed with possibly diverse layman perspectives, garnered significantly higher views. This underlines a critical facet of health information dissemination: the medium and manner of presentation are equally, if not more, paramount than the content’s clinical veracity. It beckons a pressing query: are non-physicians leveraging more relatable or engaging methods to convey their content, thus eliciting greater viewership?

Moreover, the nearly parallel DISCERN scores between these groups reiterate that the quality of information does not necessarily correlate with popularity. In the evolving digital milieu, aesthetic appeal, relatability, and presentation format play cardinal roles in determining content virality [17]. This is further emphasized by the notably higher view counts of private companies, which, while offering marginally lower DISCERN scores, seem adept at crafting visually appealing, algorithm-friendly content [18].

The surge of health information on platforms like TikTok has broader implications for patient education and treatment adherence. Historically, patient-physician interactions, brochures, and community health sessions were the cornerstone of patient education [19]. Now, with easily digestible video snippets becoming increasingly influential, there lies both an opportunity and a challenge [20]. The opportunity is to leverage this platform to enhance patient understanding, ensuring that individuals are better informed about their conditions, potential treatments, and management strategies [21]. The challenge, however, is ensuring that the tidal wave of information is both accurate and clinically sound. Misinformation can have severe repercussions, leading to inappropriate self-management strategies or misconceptions about asthma’s pathophysiology and treatment modalities [22].

Furthermore, this digital trend has implications for treatment adherence. If patients derive their knowledge primarily from social media, the perception of prescribed treatments might be influenced more by popular opinion than scientific evidence [23]. This could affect compliance with recommended therapeutic regimens, with patients potentially favoring more “viral” home remedies over evidence-based interventions. As such, healthcare professionals might need to be more proactive in addressing and debunking myths or misconceptions sourced from social media.

The temporal trend analysis of asthma-related content on TikTok between June 2023 and January 2024 reveals shifts in user engagement and content quality. Content generated by physicians and contributions from other health professionals saw a substantial rise in engagement metrics, such as views and likes, indicating increased popularity or relevance of these sources. Conversely, content from private companies experienced a notable decline in views and likes, suggesting a potential shift in audience preference or content effectiveness. Across all categories, there was a general decrease in DISCERN scores, indicating a possible reduction in the quality or reliability of asthma-related information over this period. The t-test results suggest that, statistically, there were no significant changes in views, likes, comments, favorites, and DISCERN scores between the two time points, although shares and DISCERN scores show a trend toward significance. This implies that while there were observable differences in the metrics, they were not statistically significant to confirm a definitive trend or change in the overall engagement and content quality on TikTok regarding asthma-related content. This underscores a critical challenge in digital health communication: maintaining the balance between user engagement and the accuracy and reliability of information, especially on dynamic platforms like TikTok, where content trends and audience preferences rapidly evolve. 

Limitations

This study, while comprehensive in its approach to analyzing asthma-related content on TikTok, is not without its limitations. Firstly, the reliance on the hashtag #asthma for data collection may have excluded relevant content not tagged with this specific hashtag, potentially limiting the scope of our analysis. Additionally, the focus on the English-language content only may have overlooked valuable insights from non-English speaking communities, thereby limiting the generalizability of our findings across diverse linguistic and cultural contexts.

Another key limitation is the temporal scope of the study. While the comparison of data points between June 2023 and January 2024 provides valuable insights, it covers a relatively short period in the rapidly evolving landscape of social media. Longer-term studies might reveal more significant trends and shifts in content and user engagement. Furthermore, the use of the DISCERN instrument, primarily designed for written health information, might not fully capture the nuances and specificities of video content on social media platforms. The adaptation of this tool for video content evaluation, though innovative, may not comprehensively assess the quality of multimedia elements and interactive aspects of the content. In terms of statistical analysis, while regression analysis and t-tests provide useful insights, they are limited by their inherent assumptions and may not account for all the complexities and influencing factors in social media analytics.

## Conclusions

The rise of platforms like TikTok in the health domain underscores the broader narrative of technology’s omnipresence in modern medicine. From telehealth consultations and AI-driven diagnostics to health information dissemination on social media, technology is reshaping healthcare paradigms. Our study underscores the multifaceted nature of asthma-related content on this platform, with both opportunities and challenges lying therein. While this convergence offers unprecedented opportunities, it also necessitates a structured, ethically sound framework to ensure that the fusion of health and technology remains patient-centric, evidence-based, and ethically unblemished. As the lines between traditional healthcare and digital platforms blur, it becomes imperative for stakeholders, be they physicians, health organizations, or digital platform regulators, to remain agile, adaptive, and vigilant. VHICS would provide a structured and comprehensive approach to evaluate health-related video content on social media, offering an essential tool for researchers, healthcare professionals, and even general users to assess the quality of information being disseminated on platforms like TikTok. The overarching goal must remain clear: harnessing the potential of platforms like TikTok while ensuring the sanctity and reliability of health information.
